# Associations of Masticatory Muscles Asymmetry and Oral Health with Postural Control and Leg Injuries of Elite Junior Soccer Players

**DOI:** 10.2478/hukin-2022-0086

**Published:** 2022-11-08

**Authors:** Henny Solleveld, Bram Slaets, Arnold Goedhart, Luc VandenBossche

**Affiliations:** 1 SportsInjuryLab, Dronten, The Netherlands.; 2 Physical Rehabilitation and Sports Medicine, Ghent University Hospital, Gent, Belgium.

**Keywords:** masseter muscle, temporalis muscle, electromyography, balance, sports injuries, dental caries

## Abstract

The influence of asymmetry between masticatory muscles on postural control is still under debate and only few studies examined the impact of oral health on injury risk. The present study investigated the relationships between masticatory muscles asymmetry, oral health, postural control and the prevalence of (non-contact or traumatic) leg injuries in a sample of 144 male elite junior soccer players. sEMG of the masseter and temporal muscles was performed during maximum teeth clenching, postural control was tested by measuring sway velocity during the unipedal stance with eyes closed, while oral health and the number of leg injuries were assessed using a questionnaire. The time-1 assessment was repeated in a subgroup of 69 players after one year. Pearson and partial correlation coefficients and adjusted odds ratios (OR) were used to assess associations. Asymmetry between the masseter and temporalis muscles (AMTM, quantified as anteroposterior coefficient, APC) was associated with higher sway velocity on the dominant leg (using time-1 data partial r = -0.24, p = 0.004, using longitudinal data partial r = -0.40, p = 0.005). Higher prevalence of two or more leg injuries throughout a competitive season was associated with poor oral health (adjusted OR (95%CI) using time-1 data = 2.14 (1.02–4.46), using longitudinal data = 4.47 (1.25–15.96)). These results indicate that AMTM has a negative influence on the sway velocity of the dominant leg only, possibly because frequent balancing exercises on the non-dominant leg may counteract negative influences of AMTM. The association of oral health with leg injuries underlines the need for oral health promotion and monitoring strategies in sports.

## Introduction

There is increasing recognition that postural control is a key to success in soccer and a protective factor against injuries (Hrysomallis, 2011). Postural control is regulated by a complex cybernetic system in which afferent information from the visual, vestibular and proprioceptive systems is transmitted to the central nervous system (CNS) that processes it and sends commands to the muscular system (Ivanenko and Gurfinkel, 2018). Paillard and Noe (2006) found dominance of proprioception in the postural strategy of elite soccer players, possibly because they gained the ability to perform technical maneuvers while visual attention was primarily directed at circumstances. As technical maneuvers like shooting are preferably performed with the dominant (kicking) limb, while the non-dominant (standing) limb provides stability, postural control while standing on the non-dominant leg is well developed in soccer players (Muehlbauer et al., 2019).

The functions of the postural system can be affected by several factors. Amongst others, attention has been placed on the influence of asymmetry between masticatory muscles as this asymmetry can be transmitted by muscle-facial chains to distal musculature and may influence information from the vestibular and oculomotor systems (Cuccia and Caradonna, 2009). Experimental studies have found close functional connections between masticatory, neck and trunk muscles (e.g. Giannakopoulos et al., 2013). Therefore, asymmetry between masticatory muscles may lead to asymmetrical control of the tonic muscles of the neck which may lead to asymmetrical whole-body postural control.

Another connection between the masticatory muscles and muscles activities responsible for postural control, is based on the anatomical connections between afferent neurons associated with masticatory muscles and the mesencephalic nucleus of the trigeminus (MNT). As the MNT has also a neuronal connection to the vestibular nuclei, the information from the masticatory muscles may affect the vestibular nuclei which play an essential role in maintaining postural control (Bieniek et al., 2019; Cuccia and Caradonna, 2009; Dolański et al., 2018; Gangloff and Perrin, 2002; Karimizadeh et al., 2020).

Because of these connections, a negative effect of asymmetry between masticatory muscles on postural control seems plausible, but the empirical evidence is conflicting (Julià-Sánchez et al., 2019). Possible explanations for the contradictory results include differences in the difficulty of postural tasks and in the efficiency of participants’ neurophysiological mechanisms. Our study investigated whether asymmetry between masticatory muscles of well-trained soccer players, who likely had developed effective neurophysiological mechanisms, would have a negative effect on the difficult task of single-leg standing with eyes closed on the, for single-leg standing better trained, non-dominant (standing) leg and on the less trained dominant (shooting) leg.

Gum disease and dental caries may have a negative effect on the frequency of sports injuries because both oral health problems are associated with elevated levels of cytokines, like tumour necrosis factor (TNF-a) and interleukin-6 (IL-6) (Gornowicz et al., 2012), which have an important role in the origin of muscle fatigue during exercise (Ament and Verkerke, 2009; Robson-Ansley et al., 2004). Fatigue is one of the important risk factor for soccer injuries because it deteriorates proprioception and decreases stabilizing muscle activation (Paillard, 2012). Deteriorated proprioception may trigger sports injuries, as, for example, an altered sense of the limb position may lead to the misplacement of the limb (Barrett et al., 2016). Empirical studies have found an association between poor oral health and the frequency of sports injuries of soccer players (Gay Escoda et al., 2011; Solleveld et al., 2015). In this study we tested the hypothesis that oral health problems are associated with higher prevalence of lower limb injuries during a competitive season.

Longitudinal data for this study were gathered by repeating the measurements after one year. The longitudinal data were used to investigate the temporal stability of the study variables across the year between the first (T1) and the second (T2) assessment and to replicate the cross-sectional analyses, using combined values of the first (T1) and the second (T2) assessment of the study variables because combined values are in general more reliable.

## Methods

### Participants and Procedures

The study participants included 201 not-injured players of the junior male squads of three Belgian clubs playing in the top soccer division (the Jupiler Pro League). From the initial 201 participants we excluded 57 individuals who used a fixed orthodontic appliance, leaving 144 participants in the cross-sectional sample. The one-year longitudinal study was completed by 69 participants not using a fixed orthodontic appliance and not injured at the time of the first or the second assessment. The reasons for not attending the second assessment (T2) were leaving the club (e.g., not being selected or transfer to another club), being no longer junior (i.e., older than 19 years) and being injured.

In both years, participants arrived forty-five minutes before training at the club, where they completed a questionnaire, performed single-limb-standing balance tests, and underwent a surface electromyography (sEMG) recording. The Dutch questionnaire was professionally translated into French and into English and then translated back into Dutch to ensure accuracy.

Players and their legal guardians received a written description of the research procedure and informed consent forms from their fully informed team leader(s). Parental informed consent and participants’ informed assent was obtained in the 2017-2018 pre-season. The study protocol was approved by the Ethical Committee of the Ghent University Hospital.

### Measures

#### Questionnaire variables

*Oral Health* (see Locker, 1997) was assessed by three 4-point Likert-scale questions: on the frequency of gum problems (responses ranged from 0 = never to 3 = always), on the total number of caries treatments and on the total number of removed teeth (responses ranged from 0 = zero to 3 = four or more). Questions on the total number of caries treatments and on the total number of removed teeth, have been found valid as indicators of caries problems in previous research (Gilbert et al., 2003). *Temporomandibular joint (TMJ) problems* were assessed with two 4-point Likert-scale questions (responses ranging from 0 = never to 3 = always), one on the frequency of jaw joint problems (i.e., problems with opening the mouth, clicking sounds, pain, difficulties with eating), the other on actual teeth clenching or grinding. The Oral Health and TMJ scores were computed by summing the 3 (Oral Health) and 2 (TMJ) item scores and then dichotomizing each sum score by the median value.

*Injury Frequency in the Past Year (IFPY)*. We used the broader definition of injury in the consensus statement: “Any physical complaint sustained by a player that results from a soccer match or soccer training, irrespective of the need for medical attention or time loss from soccer activities” (Fuller et al., 2006). IFPY was assessed with seven questions about how often each of the following body parts were injured in the past year: groin, hamstring, quadriceps, Achilles’ tendon, knee, ankle, and foot, e.g. “Have you had a knee injury in the past year?”. Participants responded by using a 4-point Likert scale: never = 0, once = 1, twice or three times = 2, and four times or more = 3. IFPY was computed as the sum of the seven items and dichotomised by their median value.

The determination of the *dominant leg* was based on the answer to the question about which leg is used most when kicking.

### Postural stability

We measured the postural stability of participants while they stood barefoot in a single-legged (unipedal) stance on the foot pressure platform with eyes closed. Participants were instructed to stand upright and to sway as little as possible, with the testing foot in the centre of the platform, the arms hanging loosely along the body. No instructions were given on the position of the supporting limb e.g., participants were allowed to flex the knee of the supporting limb.

Two 20-s balance trials were performed on each leg, first without then with cotton-rolls between the posterior teeth. Cotton rolls were used because they may reduce possible negative effects of malocclusion on postural stability (Tartaglia et al., 2008). The Centre of Foot Pressure (COP) displacements were assessed with the Footscan USB2-system version 7.7 (RS Scan International, Olen, Belgium) composed of a 50 x 40 cm foot pressure platform with 4096 sensors, sampling at 100 Hz. The Footscan data were input to a custom program to calculate the COP sway velocity. Postural stability on each leg was evaluated by the average COP sway velocity (mm/s) across both trials, as previous research found that sway velocity was the most reliable measure of single-leg balance (Troester et al., 2018).

### Surface Electromyography indices of masticatory muscles asymmetries

Electromyographic recordings and analyses were performed according to previous studies (Ferrario et al., 2000). Briefly, the temporalis anterior (TA) and masseter muscles (MM) of both sides (right and left) were examined using an electromyography analyser with wireless probes (BTS FREEEMG 300, BTS S.p.a., Garbagnate Milanese, Italy) and bipolar electrodes. After skin preparation with an alcohol swab, paired disposable, pre-gelled silver/silver-chloride circular electrodes with a diameter of 24 mm and an interelectrode distance of 21 ± 1 mm, (Kendall® Arbo® H124SG, Covidien, Mansfield, MA, USA) were positioned on the muscular bellies parallel to the muscular fibers.

The analog signals were amplified and digitized (gain 500, resolution 16-bit, sensitivity < 1 mV, temporal resolution 1 ms) using differential amplifiers with a high common mode rejection ratio (CMRR > 110 dB in the range 0–50 Hz, input impedance > 10 GΩ). EMG signals recorded were digitally band-pass filtered between 80 and 400 Hz with a second-order Butterworth filter and rectified by calculating the root mean square (RMS) in temporal windows of 25 ms. The system was interfaced with a computer, and BTS Dental Contact Analyzer software v2.3.20 (BTS S.p.a., Garbagnate Milanese, Italy) was used for signal recording and analysis.

First, standardization recordings that provided reference EMG values for the subsequent normalization were performed. Two 10 mm thick cotton rolls were positioned on the mandibular second premolar/first molars of each participant and a five-second maximum voluntary clench was recorded. Second, test recordings of EMG activity during a five-second MVC were performed without cotton rolls. The participant was invited to clench as hard as possible with the maxillary and mandibular teeth in maximum contact (intercuspal position), and to maintain the same level of contraction for 5 s. The 3 s with the most constant rms EMG signal of each recording were automatically selected by EMG software and used for all subsequent analyses that were automatically performed by computer software. For each muscle, the EMG potential was expressed as a percentage of the EMG potential during the standardization test (that was set at 100%). During the whole procedure, participants sat with their heads unsupported and were asked to maintain a natural erect position.

The following four *sEMG indices* were used in the present study. To assess the asymmetry between left and right jaw muscles, the EMG waves of paired (left and right) MM and TA muscles were compared by calculating a percentage overlapping coefficient (POC) that ranges between 0 (maximal asymmetry) and 100% (perfect symmetry). TA and MM POC’s (*POCTA* and *POCMM*) were obtained for each participant. To assess unbalanced contractile activity of contralateral MM and TA muscles, for instance left MA and right TA, that might give rise to a lateral displacing component, the Torque Coefficient (*TC*, unit%) was calculated. This index ranges between 0 (complete presence of lateral displacing force) and 100% (no lateral displacing force). To measure asymmetry between the masseter and temporalis muscles (AMTM), the anteroposterior coefficient (APC, unit%) was computed. The APC is 100 when standardized muscular potentials are equal and close to 0 when standardized muscle potentials are not balanced.

### Statistical analysis

To reduce the impact of extreme values, we identified outliers using the robust Median Absolute Deviation method (Leys et al., 2013). The outliers were set to the next nonoutlier value (winsorized). To correct negative skewness of the (winsorized) sEMG indices, natural logarithmic transformations were applied, using the formula NEWx = -ln(K-x), where K is equal to the largest score +2. All analyses using sEMG indices were run twice: once using the non-transformed indices and again with the transformed versions. Results were essentially unchanged when using the transformed indices. For ease of interpretation, only the results using non-transformed indices are presented.

Continuous variables are presented using means and standard deviations and categorical variables using frequencies and percentages. To assess differences between T1 and T2 data, paired T-tests were used for continuous variables and the McNemar’s test of matched binairy pairs for categorical variables. Pearson product–moment correlations were used to examine the strength and directions of the correlations between continuous variables. Partial correlation analyses were performed to explore the independent correlation between the sway velocities and each variable, controlling for all other study variables. Unadjusted (crude) odds ratios and adjusted odds ratios (adjusted for the other variables) were used to estimate the associations between leg injuries and the remaining variables.

In the analyses using longitudinal data (‘the longitudinal analyses’) we combined dichotomous T1 and T2 data using the highest of the two scores and continuous T1 and T2 data by computing the mean value. The combined values were used to replicate the analyses using T1 data (‘the cross-sectional analyses’). Temporal stability of the continuous study variables between T1 and T2 was estimated by two-way mixed-model intraclass correlation coefficients (ICC(3,1)), which indicated the absolute agreement between these study variables over the one-year period. Cohen’s kappa was used to calculate the temporal stability of the dichotomous study variables. According to Cichetti and Sparrow (1981) and Landis and Koch (1977), reliability coefficients < 0.40 were considered poor, those from 0.40 to 0.75 fair to good, and those > 0.75 excellent. The data were analyzed using SPSS software, statistical analyses were regarded as significant if the *p*-value was equal to or lower than 0.05.

## Results

Table 1 summarizes the baseline characteristics, including the distribution of the oral health items, of all participants. Participants included in the longitudinal sample (n = 69) were about 11 months younger at T1 (*p* = 0.006) then the remaining participants (n = 75).

**Table 1:** Participants’ Characteristics at T1 (n = 144).

Variables	Mean (SD) or n (%)	Variables	Mean (SD) or n (%)
**Age** (Mean, SD)	13.9 (2.1)	*Gum problems*	
**Playing position**		None	123 (85%)
Field player (n, %)	130 (90%)	(Sometimes) Present	21 (15%)
Goalkeeper (n, %)	14 (10%)	*Teeth removed*	
**Leg injuries in past year**		None	109 (76%)
0 or 1 (n, %)	89 (62%)	One	25 (17%)
2 or more (n, %)	55 (38%)	Two or more	10 (7 %)
**Sway Velocity (mm/s)**		***Dichotomized Sum OHp*.**	
- Dominant leg (Mean, SD)	74.2 (29.7)	0 or 1 (n, %)	78 (54%)
- Non-dom. leg (Mean, SD)	64.6 (25.6)	2 or more (n, %)	66 (46%)
**Surface EMG indices**		**TMJ problems**	
- POCTA (Mean, SD)	85.6 (3.6)	*Jaw joint problems*	
- POCMM (Mean, SD)	83.0 (5.6)	Never	125 (87%)
- APC (Mean, SD)	85.6 (5.5)	(Sometimes) Present	19 (13%)
- TC (Mean, SD)	89.2 (3.4)	*Teeth clenching*	
**Oral Health problems**		Never	112 (78%)
*Caries treatment*		(Sometimes) Present	32 (22%)
None	62 (43%)	***Dichotomized Sum TMJp*.**	
One time	32 (22%)	0 (n, %)	94 (65%)
Two or more times	50 (35%)	1 or more (n, %)	50 (35%)

Note: OHp . = Oral Health problems; TMJp. = TMJ problems.

Pearson correlations between the independent variables at both T1 and T2 are presented in Table 2. It may be noted that correlations between TMJ problems and Oral Health problems were significant at T1 only, while correlations between TMJ problems and asymmetry between the masseter and temporalis muscles (measured as the ACP) were significant at T1 and at T2.

**Table 2:** Pearson correlations between the independent variables at T1 (n = 144) and at T2 (n = 69).

Variables	1	2	3	4	5	6	7	8
1. Age		-.12	-.05	.00	-.06	-.05	.13	-.12
2. Playing position	^		.17*	.11	-.03	-.10	-.04	.01
3. Oral Health problems	.09	^		.18*	-.07	-.01	-.05	-.09
4. TMJ problems	-.15	^	.11		.04	-.01	-.17*	-.04
5. POCTA	-.03	^	-.20	-.18		.20*	.15	.52***
6. POCMM	.00	^	.03	.08	.23		.24**	.60***
7. APC	.19	^	-.17	-.32*	.28*	.05		.40***
8. TC	-.05	^	-.10	-.02	.62***	.49***	.34**	

Note: Correlations between independent variables at T1 are presented above the diagonal and correlations at T2 below. *: p < 0.05; **: p < 0.005; ***: p < 0.0001; ^: not computed as there were only four goalkeepers among the participants at time T2.

No significant differences between the T1 and T2 measures were found with the study variables, except for sway velocity on the non-dominant leg (eyes closed), that showed a significantly lower mean score at T2 (indicating improvement), and for TC (a sEMG indicator) that showed a significantly lower mean score at T2 (indicating a decline). Nevertheless, the temporal stability across one year of most study variables (except age) was poor. As shown in Table 3, fair to good temporal stability was found for Position (kappa = 0.85), Oral Health (kappa = 0.54), sway velocity on the non-dominant leg (ICC (3,1) = 0.51) and POCMM (ICC (3,1) = 0,43).

**Table 3:** Participants’ Characteristics at T1 and T2 (n = 69) and Intraclass Correlation Coefficients (ICC) or Cohen’s kappa for examining temporal stability of the characteristics.

	Mean (SD) or n (%)		*p*-value	ICC (95% CI) or
	T1	T2	differences	kappa
**Playing position**			1.00^1^	.85
Field player (n, %)	65 (94%)	65 (96%)		
Goalkeeper (n, %)	4 (6%)	3 (4%)		
**Leg injuries past year**			1.00^1^	.39
0 or 1 (n, %)	45 (65%	46 (67%)		
2 or more (n, %)	24 (35%)	23 (33%)		
**Oral Health problems**			1.00^1^	.54
0 or 1 (n, %)	37 (54%)	37 (54%)		
2 or more (n, %)	32 (46%)	32 (46%)		
**TMJ problems**			.50^1^	.34
0 (n, %)	49 (71%)	45 (65%)		
1 or more (n, %)	20 (29%)	24 (35%)		
**Sway Velocity (mm/s)**				
Dominant leg (Mean, SD)	63.2 (25.3)	67.9 (28.7)	.21^2^	.37 (.15 - .55)
Non-dominant leg (Mean, SD)	72.2 (26.8)	64.8 (23.3)	.02^2^	.51 (.31 - .66)
**Surface EMG indices**				
- POCTA (Mean, SD)	85.9 (3.6)	85.9 (3.3)	.96^2^	.24 (.00 - .45)
- POCMM (Mean, SD)	83.5 (5.4)	83.8 (3.7)	.61^2^	.43 (.22 - .61)
- APC (Mean, SD)	85.9 (5.4)	84.2 (7.0)	.07^2^	.31 (.08 - .50)
- TC (Mean, SD)	90.4 (1.7)	89.3 (3.0)	.00^2^	.30 (.08 - .50)

1: p-value of the McNemar’s test of matched binairy pairs; 2: p-value of the paired t-test (df = 68).

Pearson and partial correlations between independent variables and sway velocity on each leg at T1 are presented in Table 4. Sway velocity while standing on the non-dominant leg (eyes closed) was significantly associated with age (Pearson r = -0.25, *p* = 0.003; partial r = -0.28, *p* = 0.009) and the playing position (Pearson r = 0.26, *p* = 0.001; partial r = 0.26, *p* = 0.002). The negative correlation with age indicates better postural control while standing on the non-dominant leg with increasing age, while the positive correlation with the playing position indicates better postural control of field players compared with goalkeepers. Correlations with oral health, TMJ problems and sEMG indices were not significant.

**Table 4:** Pearson Correlations and Partial Correlations using T1 data relating sway velocities with age, the playing position, oral health, TMJ problems and sEMG indices.

	*Sway velocity on non-dominant (standing) leg*		*Sway velocity on dominant (shooting) leg*	
	Pearson corr. (*p*- value)	Partial corr. (*p*- value)	Pearson corr. (*p*- value)	Partial corr. (*p*-value)
Age	**-0.25** (0.003)	**-0.28** (0.009)	-0.14 (0.10	-0.05 (053)
Playing position	**0.26** (0.001)	**0.26** (0.002)	**0.25** (0.02)	**0.27** (0.002)
Oral Health problems	0.08 (0.37)	0.03 (0.76)	0.02 (0.84)	-0.01 (0.94)
TMJ problems	-0.03 (0.70)	-0.07 (0.42)	-0.08 (0.33)	-0.15 (0.08)
POCTA	0.01 (0.94)	0.04 (0.66)	0.02 (0.82)	-0.02 (0.88)
POCMM	0.08 (0.33)	0.14 (0.10)	0.10 (0.22)	0.10 (0.24)
APC	-0.02 (0.81)	0.02 (0.85)	**-0.18** (0.03)	**-0.24** (0.004)
TC	0.02 (0.82)	-0.10 (0.26)	0.10 (0.26)	0.08 (0.34)

Playing position: 0 = Field player, 1 = Goalkeeper. Partial correlations are controlled for the other variables. Bold values represent statistically significant association, p < .05

Sway velocity while standing on the dominant (shooting) leg with eyes closed showed negative Pearson and partial correlation coefficients with the APC, one of the sEMG indices (Pearson r = -0.18, *p* = 0.03; partial r = -0.24, *p* = 0.004), indicating that soccer players with higher (better) levels of the APC had lower sway velocity (i.e., better postural control) while standing on the dominant leg with eyes closed. Significant Pearson and partial correlations were found with the playing position (Table 4), correlations with age and the remaining variables were not significant.

The results of analyses of the combined T1 & T2 data were in line with the results of the analyses using T1 data. As shown in Table 5, only age was significantly associated with the overall mean sway velocity while standing on the non-dominant leg with eyes closed (Pearson r = -0.43, *p* < 0.001; partial r = -0.44, *p* < 0.001) and only the overall mean APC was significantly associated with the overall mean sway velocity while standing on the dominant leg with eyes closed (Pearson r = -0.35, *p* = 0.003; partial r = -0.40, *p* = 0.005).

**Table 5:** Pearson Correlations and Partial Correlations relating Mean of T1 and T2 sway velocities with age, and the means of T1 and T2 oral health, TMJ problems and sEMG indices

	*Sway velocity on the non-dominant (standing) leg*		*Sway velocity on the dominant (shooting) leg*	
	Pearson corr. (*p*- value)	Partial corr. (*p*- value)	Pearson corr. (*p*- value)	Partial corr. (*p*-value)
Age	**-0.43** (< 0.001)	**-0.44** (< 0.001)	-0.21 (0.09)	-0.14 (0.26)
Oral Health problems	0.14 (0.26)	0.17 (0.18)	0.07 (0.56)	-0.02 (0.91)
TMJ problems	0.13 (0.31)	-0.17 (0.18)	0.13 (0.29)	-0.16 (0.22)
POCTA	0.00 (0.99)	0.05 (0.72)	-0.04 (0.74)	0.06 (0.65)
POCMM	0,12 (0.32)	0.25 (0.052)	-0.03 (0.83)	0.05 (0.67)
APC	-0.27 (0.02)	-0.22 (0.08)	**-0.35** (0.003)	**-0.40** (0.005)
TC	-0.03 (0.79)	-0.06 (0.63)	-0.08 (0.50)	0.01 (0.98)

Partial correlation controlled for the other variables Bold values represent statistically significant association, p < .05.

The number of leg injuries in the past competitive season was associated with oral health problems in T1 data and in the combined T1 and T2 data (Table 6). Using T1 data, we found that participants with two or more oral health problems had higher odds of two or more leg injuries than participants with at most one oral health problem (crude odds ratio 1.99, 95% confidence Interval (CI) 1.01–3.94, *p* = 0.05), even after adjusting for the effects of all other variables (adjusted odds ratio 2.14, 95% CI 1.02–4.46, *p* = 0.04). The associations of the other study variables with the number of leg injuries were in the expected direction, but non-significant.

**Table 6:** Odds ratios (95% CI) for two or more leg injuries in the year before T1 (n = 144) by T1 variables and between T1&T2 (n = 69) by combined T1 & T1 variables.

Variable		Crude OR (95% CI)	Adjusted OR (95% CI)
Age	T1 data	1.15 (0.97–1.36)	1.14 (0.94–1.37)
	T1 & T2 data	1.02 (0.81– .30)	0.95 (0.71–1.29)
Playing position	T1 data	0.41 (0.11–1.54)	0.28 (0.06–1.29)
Goalkeeper^	T1 & T2 data	Not Appl. (4 goalk.)	Not Appl. (4 goalk.)
Oral Health problems	T1 data	**1.99 (1.01–3.94)**	**2.14 (1.02**–**4.46)**
2 or more^	T1 & T2 data	2.33 (0.82–6.58)	**4.47 (1.25**–**15.96)**
TMJ problems	T1 data	1.65 (0.82–3.33)	1.61 (0.74–3.54)
1 or more^	T1 & T2 data	0.62 (0.15–2.54)	0.37 (0.07–1.91)
Sway velocity of the	T1 data	1.00 (0.99–1.01)	0.99 (0.98–1.01)
dominant leg	T1 & T2 data	1.00 (0.98–1.02)	0.98 (0.95–1.02)
Sway velocity of the	T1 data	0.99 (0.98–1.01)	1.01 (0.99–1.03)
non-dominant leg	T1 & T2 data	0.99 (0.97–1.01)	1.01 (0.98–1.04)
POCTA	T1 data	0.92 (0.84–1.01)	0.94 (0.83–1.06)
	T1 & T2 data	1.10 (0.91–1.34)	1.08 (0.84–1.40)
POCMM	T1 data	0.97 (0.91–1.03)	0.98 (0.90–1.06)
	T1 & T2 data	0.97 (0.81–1.14)	0.90 (0.71–1.14)
APC	T1 data	0.96 (0.90–1.02)	0.97 (0.90–1.05)
	T1 & T2 data	1.03 (0.93–1.14)	0.99 (0.87–1.14)
TC	T1 data	0.91 (0.82–1.00)	0.99 (0.84–1.16)
	T1 & T2 data	1.16 (0.83–1.62)	1.38 (0.81–2.35)

Note: goalk. = goalkeepers; ^: Reference category of the Playing position is the Field player, of Oral Health problems is 0 or 1 problems; of TMJ problems is 0 problems. ORs in **bold** are significant, p < 0.05.

Using the combined T1 & T2 data, we found a trend toward higher odds of two or more leg injuries in the year between T1 and T2 among participants with an overall mean of two or more oral health problems (crude odds 2.42, 95% CI - .87–6.75, *p* = 0.09), but significantly higher odds were found after adjusting for the effects of all other variables (adjusted odds 4.47, 95% CI 1.25– 15.96, *p* = 0.02). No associations with leg injuries were found for the other variables.

## Discussion

The current study examined the associations of imbalance in the masticatory muscles, oral health and TMJ problems with postural control and with frequency of leg-injuries of elite junior soccer players. In the analyses using T1 data and in the analyses using T1 & T2 data, we found that (1) higher sway velocity while standing on the dominant leg was associated with more asymmetry between the activities of the masseter and temporalis muscles (APC), but not with age, (2) higher sway velocity while standing on the non-dominant leg was associated with lower age, but not with the APC. Poor oral health was associated with a higher frequency of two or more leg injuries throughout a competitive season.

The finding that asymmetry between the masseter and temporalis muscles was differentially correlated with sway velocity of the dominant and the non-dominant leg, indicates that neurophysiological mechanisms that may reduce the influences of this asymmetry on postural control on the dominant leg, are not effective enough while the reverse is true for the non-dominant leg. This difference cannot be explained by differences in postural control between the legs, as nearly identical levels were found in this and previous research (e.g., Gstöttner et al., 2009). Instead, a difference in the efficiency of neurophysiological adaptations may be explained by differences in the volume of balance-training. Soccer players perform more balance training on the non-dominant leg because they use the non-dominant leg as a standing leg in passing, kicking, and landing (Muehlbauer et al., 2019). The finding that age was negatively associated with sway velocity on the non-dominant leg, but not associated with sway velocity on the dominant leg, most likely also reflects the effect of more balance training on the non-dominant leg.

Previous studies have found that balance training may lead to improvement in motor systems controlling muscular output and adaptations in visual, vestibular and proprioception systems that assist postural control (e.g., Fernández-Rio et al., 2019; Hrysomallis, 2011). Taube et al. (2008) found that balance training may lead to highly task-specific neural adaptations at different sites of the central nervous system. Thus, differences in balance training on the dominant and the non-dominant leg may lead to different neural adaptations for the legs.

The importance of balance training for the development of neural adaptations is supported by the results of a study by Maitre and Paillard (2016) on the influence of vestibular manipulation conditions (i.e., transmastoïdal GVS at 0.5 mA and 3 mA) on postural control of (1) a group of sport science students who have practiced sport at (sub) elite level, and (2) a group of individuals who have not practiced any sport activities for at least three years. The vestibular manipulation lowered postural control of the second group, but not of the first one. The authors note that the GVS signal triggers an inappropriate response because it is interpreted by the CNS as a real head rotation. In participants who practiced sports activities the CNS generates adaptive postural adjustments to withstand the erroneous signal. The practice of sport activities might have contributed to the development of adaptation and habituation mechanisms that reduce the effects of the erroneous signal. Also, sport activities may contribute to a better use of other available sensory information (i.e., proprioceptive and cutaneous sensory information).

We found that the APC, but not the other sEMG indices, showed an association with sway velocity on the dominant leg. Previous studies found that the APC was the only sEMG index associated with myogenous TMD (Mapelli et al., 2016). In this study we found associations with self-reported TMJ problems. As Nota et al. (2017) showed that myogenous TMD was associated with higher levels of postural sway, we may tentatively suggest that the association between the APC and sway velocity can also be partly explained by the presence of myogenous TMD.

The association between poor oral health and leg injuries in the cross-sectional and longitudinal analyses is consistent with the results of previous studies (Gay Escoda et al., 2011; Solleveld et al., 2015). Although only a moderate association was found, as elite athletes have a high risk of caries and periodontal disease due to stress and the use of an acidic diet, this finding underlines the importance of oral health in sports.

This study’s findings are subject to several limitations. First, self-report information is common in studies using large samples, but response bias (i.e., denial of vulnerability), recall error, and lack of insight, may have influenced the results. However, good agreement was shown between self-reported and professionally assessed oral health (Gilbert et al., 2003) and in a preliminary study we found good agreement between numbers of self-reported and registered leg injuries. Second, only static postural stability was examined. As the correlation between static and dynamic postural stability is low (Pau et al., 2015), using measures of dynamic postural stability may have resulted in different findings. Third, participants included in this study were elite junior male soccer players, thus, care is needed when generalizing the findings. Finally, the longitudinal analysis is limited by the sample size.

## Conclusions

The current study, using cross-sectional and longitudinal data from elite junior male soccer players, found that asymmetry between the masseter and temporalis muscles was associated with higher sway velocity while standing on the dominant leg, but not with sway velocity while standing on the non-dominant (standing) leg. This finding provides evidence for both the association between imbalance in the masticatory muscles and postural control and the modulating influence of neurophysiological adaptations (induced by practice and training) on this association. This study also found that oral health problems were associated with higher numbers of leg injuries in elite junior male soccer players, even after controlling for age, the player’s position, four indices of masticatory asymmetry, self-reported TMJ problems and sway velocity of each leg. These results underline the need for oral health promotion and monitoring strategies in sports.
